# Myosuppressin is involved in the regulation of pupal diapause in the cabbage army moth *Mamestra brassicae*

**DOI:** 10.1038/srep41651

**Published:** 2017-01-31

**Authors:** Nobuto Yamada, Hiroshi Kataoka, Akira Mizoguchi

**Affiliations:** 1 Division of Biological Science, Graduate School of Science, Nagoya University, Nagoya, Japan; 2 Department of Integrated Biosciences, Graduate School of Frontier Sciences, The University of Tokyo, Kashiwa, Chiba, Japan

## Abstract

Diapause, a programmed developmental arrest, is common in insects, enabling them to survive adverse seasons. It is well established that pupal diapause is regulated by ecdysteroids secreted by the prothoracic glands (PGs), with cessation of ecdysteroid secretion after pupal ecdysis leading to pupal diapause. A major factor regulating the gland activity is prothoracicotropic hormone (PTTH) secreted from the brain. In our previous study, we demonstrated that the cessation of PTTH release after pupal ecdysis resulted in the inactivation of the PGs, leading to pupal diapause in the cabbage army moth *Mamestra brassicae*. Here we show that a neuropeptide myosuppressin also contributes to the inactivation of PGs at the initiation of diapause. Myosuppressin suppresses PTTH-stimulated activation of the PGs *in vitro*. Concentrations of myosuppressin in the hemolymph after pupal ecdysis are higher in diapause pupae than in nondiapause pupae.

Insects have developed a variety of strategies in order to adapt to the conditions of their habitat. For example, many species enter diapause, a programmed developmental arrest, to survive a season(s) unsuitable for their life. Insects enter diapause at various developmental stages, i.e., egg, larva, pupa, and adult, but the stage at which diapause occurs is fixed in each species. Diapause in some insects is genetically programed (obligatory diapause), but in others is regulated by environmental conditions (facultative diapause). In the latter case, diapause is generally induced by seasonal changes in day length and terminated by the experience of low temperature for a certain period of time^1^,^2^. It is well established that diapause is regulated by hormones. The key hormone involved in larval and pupal diapause is the molting hormone, ecdysteroid, that is secreted by a pair of prothoracic glands (PGs) typically located in the prothorax of insects^3^,^4^. Since ecdysteroids are responsible for the induction of molting and metamorphosis, the arrest of their secretion is considered to be a major cause of the initiation of diapause. In fact, in diapausing larvae and pupae, the ecdysteroid titers in the hemolymph are very low^5^,^6^,^7^,^8^. It is well known that the activity of the PGs is regulated by prothoracicotropic hormone (PTTH) produced by the neurosecretory cells in the brain^9^,^10^. Thus, there has been a long-standing belief that the cessation of PTTH secretion leads to the initiation of diapause since Carroll Williams proposed this hypothesis in 1952 based on his study using diapause pupae of the giant silk moth *Hyalophora cecropia*^11^. However, this hypothesis was verified only recently by demonstrating the absence of PTTH in the hemolymph of diapause pupae in the cabbage army moth *Mamestra brassicae*^12^, although indirect evidence for the decline of PTTH secretory activity of the brain in diapausing pupae had previously been presented in some insects^3^. In *M. brassicae*, pupal diapause is induced by short day conditions as with many other insects, and the diapause pupae do not start pupal-adult development for months when kept at room temperature. In diapause-destined final instar larvae of *M. brassicae*, the PTTH titer in the hemolymph has been shown to decrease after the onset of wandering, as compared with non-diapause animals, and after pupal ecdysis no PTTH is detected in the hemolymph, in contrast to high PTTH titers in the hemolymph of non-diapause pupae. These observations have suggested that the cessation of PTTH secretion leads to pupal diapause, and this idea was supported by the demonstration that injection of PTTH to the newly ecdysed diapause-destined pupae prevented them from entering diapause^12^. In some cases of larval diapause, the involvement of juvenile hormone (JH) in the maintenance of diapause has been suggested^13^,^14^.

In our study in 2013, however, we noticed that PTTH injection to diapause-destined pupae became much less effective only one day after pupal ecdysis, and this interesting observation prompted us to investigate the mechanisms underlying a rapid decline in PTTH responsiveness of early diapause pupae. In this report, we demonstrate that myosuppressin, one of the recently discovered prothoracicostatic neuropeptides, is involved in this mechanism. Neuropeptides that inhibit PG activity *in vitro* have been identified in the silkmoth *Bombyx mori*. The first reported PG-inhibitory neuropeptide was the prothoracicostatic peptide (PTSP), which is structurally related to insect myoinhibiting peptides (MIPs) and allatostatin-B^15^. In *B. mori* and the tobacco hawkmoth *Manduca sexta*, PTSP is secreted from the central nervous system (CNS) and more abundantly from the epiproctodeal glands, and is thought to suppress ecdysteroid secretion by the PGs at the end of each molting cycle^16^. The second PG-inhibitory neuropeptide is *B. mori* myosuppressin (BMS), which is structurally related to known insect myosuppressins and which exerts strong PG-inhibitory action to the PG in *B. mori*^17^. BMS is expressed widely in the nervous system, but remarkably two pairs of BMS-producing neurosecretory cells in the brain project their axons to the corpora cardiaca (CC) and nervi corporis cardiaci-recurrence (NCC-RN), where a large amount of BMS is accumulated^17^. The BMS gene expression in the brain is high during the feeding period of the fifth instar but is low after the onset of wandering, suggesting its role in the regulation of the timing of metamorphosis. The third PG-inhibitory neuropeptide is BRFa, a member of FMRFamide-related peptides (FaRPs), predominantly expressed in neurosecretory cells of the thoracic ganglia in *B. mori*. This peptide is not released into the hemolymph but delivered via axons from the prothoracic ganglion to the PGs, acting directly on the PG cells^18^. This peptide is also considered to regulate the timing of metamorphosis by suppressing the PG activity.

Although all these neuropeptides are suggested to play roles in the regulation of PG activity during larval-pupal development in *B. mori*, their function as developmental regulators has not yet been fully established, due to a lack of demonstrations of their *in vivo* actions and of information about their hemolymph titers, and to a paucity of studies in other insects and at other developmental stages. Here, we show that BMS is involved in the regulation of PG activity in *M. brassicae*, playing an important role in the initiation and maintenance of pupal diapause.

## Results

### Developmental changes in the effect of PTTH injection into diapause pupae

**Figure 1:**
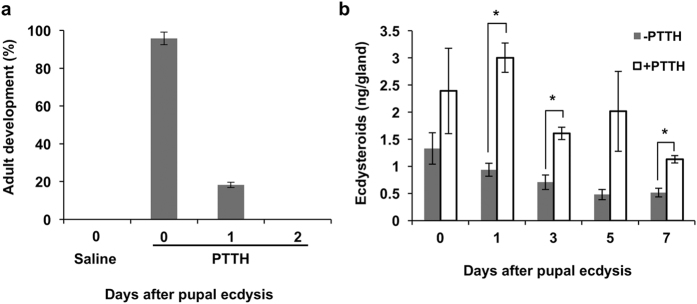
Effects of PTTH on the activation of PGs in diapause pupae. (**a**) Crude PTTH was injected into diapause pupae 0, 1 or 2 days after pupal ecdysis, and the percentage of the pupae that started adult development was calculated. As a negative control, Ringer was injected within 6 h after pupal ecdysis. The values shown are the means (±SEM) of three independent determinations (n = 6–12). (**b**) PGs were dissected from 0–7 days after pupal ecdysis. They were incubated in the presence or absence of PTTH for 3 h, and the amount of secreted ecdysteroids was determined by ELISA. The values are the means (±SEM) of six independent determinations. Student’s t-test, *P < 0.05.

We previously demonstrated that the cessation of PTTH secretion after pupal ecdysis was critical for the initiation of diapause. One piece of the evidence leading to this conclusion was that the injection of PTTH into newly ecdysed diapause pupae was able to induce adult development^12^. Intriguingly, however, the injection of PTTH was only effective when given within a day after pupal ecdysis: The next day, the response of pupae to PTTH injection was very limited, and no response was observed two days after ecdysis (Fig. 1a). These results suggested that the PGs of diapause pupae rapidly lose their responsiveness to PTTH soon after pupation. The above results might also be interpreted as a rapid loss of the responsiveness of peripheral tissues to secreted ecdysteroids. This is unlikely, however, because the injection of 20-hydroxyecdysone to day-2 diapause pupae successfully induced adult-development (data not shown).

### Changes in the responsiveness of the PGs to PTTH *in vitro*

To investigate the changes in the sensitivity of the PGs to PTTH, we collected the PGs at various times after pupal ecdysis and determined their responsiveness to PTTH *in vitro*. One of a pair of PGs from a pupa was incubated in the medium without PTTH to determine the basal ecdysteroid secretory activity of the gland. The other was incubated in the presence of PTTH to estimate the PTTH sensitivity of the gland. While the basal secretory activity of the gland gradually decreased, the PTTH sensitivity was maintained for at least a week (Fig. 1b). This result indicated that the PGs of diapause pupae retain the potential to respond to PTTH for at least several days after pupal ecdysis. This observation contradicts the previous results that the PGs *in vivo* rapidly lose their sensitivity to injected PTTH, leading us to hypothesize that the PGs *in vivo* are somehow inhibited from responding to PTTH shortly after pupal ecdysis.

### Negative regulation of PG activity by the central nervous system

In previous studies in *B. mori*, several kinds of neuropeptides that inhibit the activity of PGs to secrete ecdysteroids have been identified^15^,^17^,^18^. Therefore, we supposed that one of these factors could suppress the response of the PGs to PTTH at this stage. To verify this hypothesis, we examined the effects of these factors on the PGs using an *in vitro* culture system.

**Figure 2:**
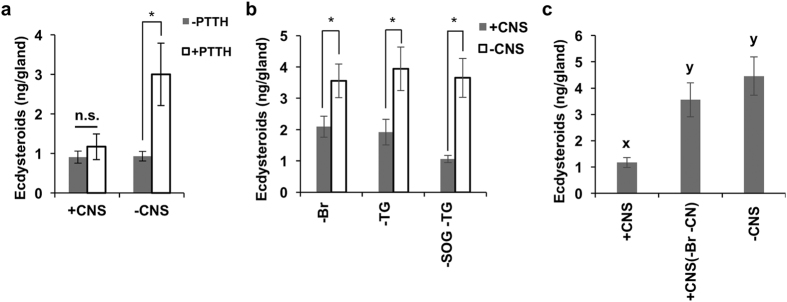
Suppression of PTTH-stimulated activation of the PGs by the CNS. (**a**) PG preparations with or without CNS were incubated in the presence or absence of PTTH for 3 h. The amount of secreted ecdysteroids was determined by ELISA. The values are the means (±SEM) of six independent determinations. (**b**,**c**) One or two ganglia (**b**) or both the brain and connected nerves (**c**) were removed from the CNS. Br, brain; TG, thoracic ganglion 1; SOG, suboesophageal ganglion; CN, connective nerves. The PGs with truncated CNS were incubated in the presence of PTTH for 3 h. The values of secreted ecdysteroids are the means (±SEM) of six independent determinations. Statistic analysis was performed using Student’s t-test (**a**,**b**) or Tukey-Kramer multiple comparison test (**c**). *P < 0.05. Different letters above the bars indicate a significant defference.

We prepared PGs with or without CNS as described in the Methods section, because the above-mentioned PG-inhibitory peptides were all derived from the CNS. Both types of PGs were incubated with or without PTTH for 3 h, and the amount of secreted ecdysteroids was determined by ELISA (Fig. 2a). When the PG was incubated without CNS, ecdysteroid secretion by the PG was significantly increased by the addition of PTTH. However, when the PG was incubated with CNS, ecdysteroid secretion was not stimulated by PTTH at all.

These results strongly suggested that the CNS inhibited a PTTH-stimulated activation of the PGs. Therefore, in order to identify the source of an inhibitory factor involved, we next incubated the PG preparation with a CNS lacking one or two of three ganglia, the brain, suboesophageal ganglion and thoracic ganglion 1, in the presence of PTTH (Fig. 2b). If the removed ganglia are the source of the inhibitory factor, the cultured PGs should respond to PTTH. In all cases, however, PGs were not stimulated by PTTH. One possible interpretation of this result is that the PG-inhibitory factor is stored outside the CNS. This hypothesis did not contradict our knowledge about the distribution of the known PG-inhibitory neuropeptides, especially that of BMS, because in *B. mori* this peptide is produced by two pairs of neurosecretory cells in the brain and a significant amount of BMS is stored in the neurohaemal organs including the CC and NCC-RN^17^. Therefore, we next removed the brain and the neurohaemal organs from the PG preparation with CNS and incubated the PG with PTTH. In this case, the PG was significantly activated by PTTH (Fig. 2c). This result strongly suggested that a BMS-like peptide is involved in the regulation of PG activity in early diapause pupae of *M. brassicae*.

### Identification of myosuppressin in *M. brassicae*

**Figure 3:**
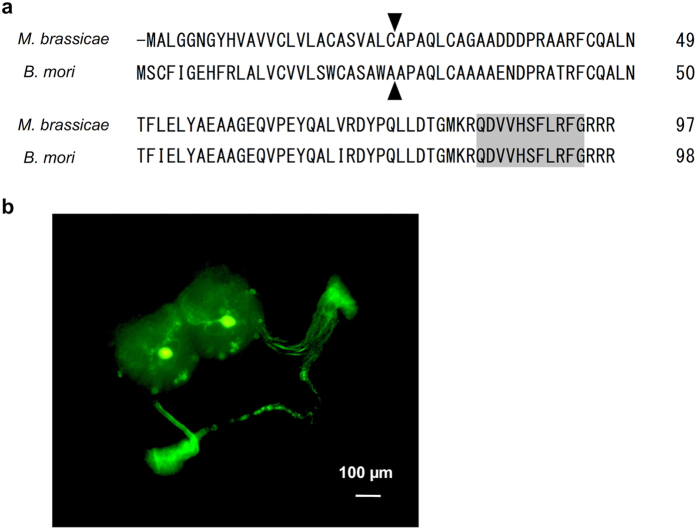
Identification of *M. brassicae* myosuppressin. (**a**) Comparison of the amino-acid sequences of the precursor peptides for *M. brassicae* and *B. mori* MSs. The shaded sequences denote predicted mature peptides. The predicted signal peptide cleavage for MS precursors is indicated with an arrowhead. (**b**) Whole-mount immunohistochemistry on the day-1 sixth instar larval brain and connected tissues with an anti-BMS mouse monoclonal antibody.

To clarify whether BMS or a BMS-like peptide regulates the PGs in diapause pupae, we first cloned the myosuppressin gene of *M. brassicae* and determined the nucleic acid sequence of the gene. The deduced open reading frame encoded 98 amino acid residues, and this precursor peptide showed high homology (78%) to preproBMS and a predicted mature peptide was identical to BMS (Fig. 3a). Immunohistochemistry using anti-BMS antibody revealed that *M. brassicae* myosuppressin (MS) is produced by two pairs of neurosecretory cells in the brain and stored in large quantities in the CC and NCC-RN, like BMS in *B. mori* (Fig. 3b).

### The effect of MS on the activity of PGs

**Figure 4:**
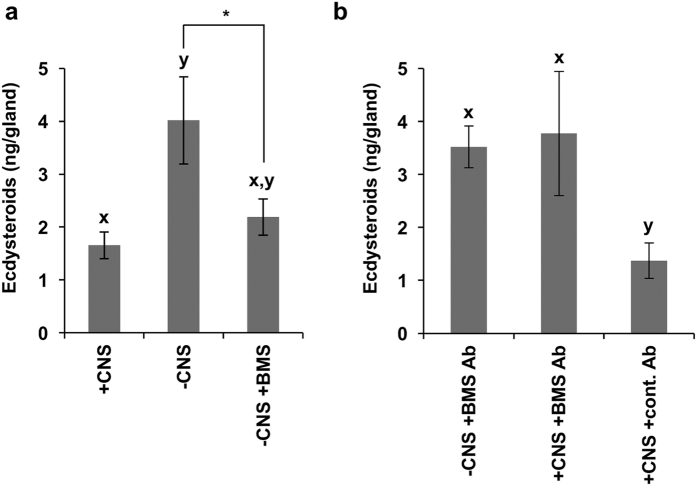
Suppression of PTTH-stimulated activation of the PG by BMS. (**a**) The PG preparation without CNS (−CNS) was incubated for 3 h in the presence of only PTTH or both PTTH and BMS. For comparison, the PG with CNS (+CNS) was also incubated in the presence of only PTTH. The amount of secreted ecdysteroids was determined by ELISA. (**b**) The PG preparation with CNS (+CNS) was incubated for 3 h in the presence of both PTTH and anti-BMS antibody (BMS Ab). For comparison, the PG without CNS (−CNS) was also incubated in the same way. In the control, the anti-BMS antibody was replaced by an unrelated antibody (cont. Ab). The values are the means (±SEM) of six independent determinations. Different letters above the bars indicate a significant difference (ANOVA followed by Tukey-Kramer multiple comparison test). A specific pair of values in (**a**) was further compared using Student’s t-test. *p < 0.05.

We then examined the effect of MS on the activity of the PGs of diapause pupae in two ways using an *in vitro* culture system. Since *M. brassicae* MS was identical to *B. mori* MS (BMS), an existing BMS peptide and anti-BMS antibody^17^ were used in these experiments. When only PTTH was added to the culture of the PG preparation without CNS, the PG was activated, as in the previous experiments (Fig. 4a). In contrast, when BMS was added together with PTTH to the culture, the PG was not activated (Fig. 4a).

If MS is secreted from the CNS to suppress PG activation, this effect of MS could be blocked by addition of anti-BMS antibody to the culture. To examine this possibility, the antibody was added together with PTTH to the cultured PG preparation with CNS. As a result, ecdysteroid secretion was significantly increased as compared to the control, where the BMS antibody was replaced with an unrelated antibody (Fig. 4b).

These results strongly suggested that MS is secreted at the initiation of pupal diapause to suppress the activation of PGs.

### Developmental changes in hemolymph MS titers and MS gene expression in the brain

**Figure 5:**
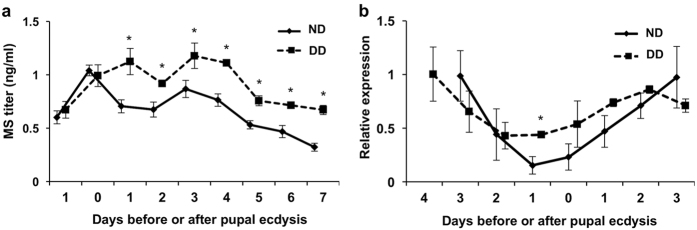
Developmental changes in the hemolymph MS titers and in *MS* gene expression in the brain. (**a**) The MS titers in the hemolymph of nondiapause (ND)- and diapause-destined (DD) insects were determined by TR-FIA. (**b**) Relative expression levels of the *MS* gene in the brains of ND and DD insects were determined by qRT-PCR. The *MS* transcript levels were normalized with the *RpL8* transcript levels in the same samples and are indicated in relative values, with the highest value being 1.0. The values shown are the means (±SEM) of eight (**a**) or three (**b**) independent determinations. Student’s t-test, *P < 0.05.

In order to examine if MS is actually secreted at the initiation of pupal diapause, we investigated the developmental changes in the hemolymph MS titer. For this purpose, we developed a new assay method to measure hemolymph MS titers. *M. brassicae* MS was extracted from the hemolymph with acid methanol and the peptide was quantified by a time-resolved fluoroimmunoassay (TR-FIA) using anti-BMS antibody and a competitive assay protocol. The hemolymph MS titers were determined over a period of one day before pupal ecdysis to 7 days after ecdysis. From one day after pupal ecdysis onward, MS titers in diapause-destined pupae were significantly higher than those of nondiapause-destined pupae (Fig. 5a). This result supported our hypothesis that MS is involved in the suppression of PG activity in diapause pupae.

Next, we measured the *MS* mRNA levels in the brain of nondiapause- and diapause-destined insects by quantitative RT-PCR (qRT-PCR) over a period from the day of wandering to three days after pupal ecdysis. Expression of the *MS* gene tended to be higher in diapause-destined insects than in nondiapause-destined ones for several days around pupal ecdysis, although significance was only noted one day before pupal ecdysis (Fig. 5b), consistent with our observation that the hemolymph MS titers were higher in diapause-destined animals.

### The effect of anti-BMS antibody injection into early diapause pupae

To confirm the effect of MS in inhibiting the response of the PG to PTTH in early diapause pupae, we injected anti-BMS antibody into day-0 diapause pupae and one day later injected PTTH into the same animals. If the antibody could block the action of MS on the PGs, the glands should be activated by PTTH. However, pupae did not start adult development, suggesting that the PG did not respond to PTTH.

## Discussion

In this study, we demonstrated that MS appears to regulate PG activity in the initiation of pupal diapause of *M. brassicae*. MS was first identified in the cockroach *Leucophaea maderae* as a peptide that reduces the frequency of hindgut contractions^19^ and then found in a wide variety of insects^20^. MS generally shows inhibitory activity against various kinds of visceral muscles of insects but has a prothoracicostatic action in *B. mori*^17^. In *B. mori*, MS (referred to as BMS) suppresses both the basal and PTTH-stimulated ecdysteroidogenesis by the PG *in vitro* through inhibition of cAMP accumulation in the gland^17^. Although the PG-inhibitory action of BMS is evident, the physiological role of this peptide in the regulation of *Bombyx* development remains unclear. In the present study, we demonstrated its prothoracicostatic activity in *M. brassicae* and suggested its role in the regulation of insect diapause. Thus, this is the second report to show the prothoracicostatic action of MS in insects and the first report to suggest a physiological role in the regulation of insect diapause. To our knowledge, no other factors have been identified that suppress PG activation at the initiation of pupal diapause.

In our previous study, we demonstrated that the cessation of PTTH secretion after pupal ecdysis induces diapause^12^. Although this change must be the primary cause of the initiation of pupal diapause, a stable inactivation of the PG for deeper diapause appears to require an active inhibition of the gland, in addition to a passive inhibition by the absence of PTTH. In our PG culture system, the CNS or exogenous BMS strongly inhibited PTTH-stimulated ecdysteroidogenesis but not basal levels of ecdysteroidogenesis at all, suggesting that MS inhibits the signal transduction pathway of PTTH in PG cells. This action of MS slightly differs from that of BMS observed in *B. mori*^17^, because in *B. mori* BMS suppressed both the basal and PTTH-stimulated ecdysteroidogenesis. However, this difference might be attributed to the difference in the PG culture system. The PG was incubated together with the surrounding tissues in the present study, while the gland was isolated before incubation in the *Bombyx* study. The isolated PG secretes much more ecdysteroids than the PG dissected together with nearby tissues^21^, suggesting that the stress of isolation of the PG may also stimulate the PG. If this is the case, the effect of BMS on basal ecdysteroidogenesis observed in the previous study may also be an inhibition of stimulated ecdysteroidogenesis.

If the role of MS in the regulation of diapause is to inhibit the activation of steroidogenesis by the PGs, it is unclear why this peptide is released after the shutdown of PTTH secretion. One possible reason for MS secretion is to prevent the activation of the PG by an accidental release of PTTH from the brain. However, another interpretation may also be possible. Although PTTH is the major PG tropic hormone, some other hormones or neuropeptides have also been reported to stimulate the PGs in some specific conditions. Among them are bombyxin or insect insulin^22^,^23^, diapause hormone^24^,^25^, orcokinin^26^, and juvenile hormone^27^,^28^. If some of these factors are normally or accidentally released in diapausing pupae, it must be a critical demand to prevent the activation of the PGs by them. Therefore, MS might be released after pupal ecdysis to keep the PGs inactive in the presence of these peptides. Whether or not MS inhibits the action of these peptides on the PGs must be investigated in the future.

It is also interesting to speculate about the role of MS in the maintenance and termination of diapause. In our previous study, we demonstrated that PTTH secretion and consequent PG activation is necessary for the initiation of post-diapause development^29^. We expect MS secretion level to be relatively high during diapause but low after diapause termination. Our study on the endocrine mechanisms regulating diapause maintenance and termination, which includes the measurement of MS concentrations in the hemolymph, is under way.

We have developed an assay for measuring MS and have succeeded in determining its titers in the hemolymph. This is the first report describing the concentration of MS in the hemolymph. It was around 1 nM, and was higher in diapause pupae than in nondiapause ones for a week after pupal ecdysis (Fig. 5a). It is interesting to note that this concentration of MS corresponds to its optimal concentration determined in a previous study in *B. mori*, where it was most effective at around 10^−9^ M and less effective at higher or lower concentrations^17^. However, the difference in the titers between the two types of pupae in *M. brassicae* was smaller than expected. It is unlikely that this small difference is critical for the PG-inhibiting action of MS, because the effect of BMS was almost the same between the concentrations of 10^−10^ and 10^−9^  M^17^. It is possible that the MS receptor is expressed at much higher levels in diapause pupae. However, we prefer the following explanation: The 1 nM concentration of MS may be high enough to suppress the activation of the PG of diapause pupae, because in diapausing pupae the stimulus by PG tropic factors, if any, may be weak. In contrast, in nondiapause pupae the presence of MS at lower concentrations may be ineffective to completely suppress PG activity due to a high concentration of PTTH in the hemolymph^12^. In fact, the *Bombyx* PG *in vitro* responded to PTTH in a dose-dependent manner even in the presence of 1 nM BMS, although the secreted amount of ecdysteroids was reduced as compared to that in BMS-free conditions^17^. It is likely that the activity of the PG may be regulated by the balance between stimulation by PTTH and inhibition by MS.

We cannot preclude the possibility that other factors also contribute to the inactivation of the PGs in diapause pupae. For instance, it is suggested that PTSP (or MIPs) is massively released from the epiproctodeal glands at the end of each molting cycle^16^. If this peptide is continuously released even after pupal ecdysis, it might inhibit the reactivation of the PGs effectively. BRFa also has prothoracicostatic activity in *B. mori*. However, its role in suppressing PG activity in the initiation phase of diapause may be very limited, if any, because removal of thoracic ganglion 1, the sole organ supplying this peptide to the PGs, did not affect the PTTH refractoriness of the PG co-cultured with CNS (Fig. 2b). Another known PG-inhibitory factor is juvenile hormone (JH). JH has been demonstrated to inhibit the activation of the PGs in the early stages of the final larval instar^21^,^27^,^30^. At present, however, no information is available on its role in the initiation of pupal diapause.

Although addition of anti-MS antibody to the PG culture successfully blocked the effect of MS (Fig. 4b), the injection of both MS antibody and PTTH to the newly ecdysed diapause pupae failed to break their diapause. This result does not necessarily negate our hypothesis that MS is released in early diapause pupae to inhibit the activation of the PGs by PTTH and/or other tropic factor(s), because blocking the effect of a hormone by a corresponding antibody *in vivo* is difficult in our experience possively due to the diffusion and/or inactivation of the antibody. An alternative approach to test our hypothesis *in vivo* is gene knockout or knockdown of *MS*. However, RNAi, especially larval RNAi, is quite difficult in lepidopteran insects^31^. We have tried to knockout the *MS* gene using a CRISPR/Cas 9 system. However, no mutant lines lacking the *MS* gene have been established. Since *MS* is expressed in a variety of tissues including the CNS and midgut^20^, the mutant animals may be embryonic lethal. Thus, at present it is difficult to verify our hypothesis by *in vivo* experiments. However, considering the clear *in vitro* effect of MS and significantly higher hemolymph titers of MS in early diapause pupae, we believe that this neuropeptide plays an important role(s) in the initiation and maintenance of pupal diapause in *M. brassicae*.

In this species, the PGs themselves appear to remain responsive to PTTH for at least a week after pupal ecdysis even in diapause-destined animals (Fig. 1b). However, this may not be the case in some other insects. For example, the PG of diapause-destined *M. sexta* pupae just after ecdysis exhibited much lower responsiveness to PTTH *in vitro* as compared to non-diapause pupae^32^, suggesting that in this species the PGs may autonomously become refractory to PTTH after pupal ecdysis. Thus, the endocrine mechanism by which the initiation of pupal diapause is regulated may vary between species.

Ecdysteroids are the key hormone in the regulation of not only pupal diapause but larval diapause as well. In both cases, the hemolymph ecdysteroid titer is kept very low during diapause. So far, the main cause of PG inactivation during diapause has been thought to be the arrest of PTTH secretion, although some aspect of larval diapause was ascribed to a high JH titer inhibiting the activation of the PGs. This may be true, but the present study strongly suggests that we should pay more attention to prothoracicostatic neuropeptides including MS for understanding the mechanisms that deepen and maintain the diapause state of insects.

## Materials and Methods

### Animals

Eggs of *M. brassicae* were obtained from a laboratory colony maintained at the National Institute of Agrobiological Sciences, Japan. Larvae were reared on an artificial diet “Insecta LFS” (Nihon Nosan Kogyo, Yokohama, Japan) at 25 °C under a 14-h light: 10-h dark photoperiod (long-day conditions) or at 23 °C under a 10-h light: 14-h dark photoperiod (short-day conditions). The animals under the long-day and short-day conditions entered pupal diapause at rates of 0% and 100%, respectively.

### Antibodies

Anti-BMS mouse monoclonal antibody was produced in our laboratory^17^. Anti-ecdysone antiserum was the same as that used in our previous studies^12^,^29^. Horseradish peroxidase-labeled anti-rabbit IgG antibody, Alexa Fluor 488-labeled goat anti-mouse IgG antibody, and biotin-labeled anti-rabbit IgG antibody were purchased from Thermo Fisher Scientific, Life Technologies and Amersham Biosciences, respectively.

### Preparation and injection of crude PTTH

Crude PTTH was prepared as described previously^12^. Briefly, the homogenate of larval brains was heated at 70 °C for 5 min, and the supernatant after centrifugation was concentrated by ultrafiltration over 10 kDa cut-off membrane. The PTTH titer of the condensed brain extract was quantified by time-resolved fluoroimmunoassay (TR-FIA) as an amount relative to that contained in a brain of the day-1 wandering larva, which was defined as 1 unit (U) of PTTH. The brain extract (crude PTTH) was injected as described previously^12^.

### *In vitro* culture of the PGs

PG culture was performed in two ways. When isolated PGs were used, they were dissected from pupae as described previously^12^ and preincubated for 1 h in Grace’s Insect Medium (Sigma). The glands were cultured for 3 h at 25 °C in the medium (200 μl) with or without 1 U of PTTH. When the PGs were incubated with the CNS, the PG was taken together with surrounding tissues including the CNS. Specifically, the pupa was cut at the position between the prothorax and mesothorax to obtain the anterior end of the body containing a pair of PGs and a nerve cord including the brain and suboesophageal and prothoracic ganglia. Then the dorsal part of this piece was removed, and the ventral part was further cut into two pieces along midline so that one half of the piece contains the PG and the nerve cord and the other half contains only the PG. The latter was use as a control PG without CNS.

### Determination of ecdysteroid content in the culture medium by enzyme-linked immunosorbent assay (ELISA)

The culture medium after PG incubation was diluted 10-fold with 0.5% bovine serum albumin in TBS (50 mM Tris-HCl, 0.9% NaCl, pH 7.8) and subjected to ecdysteroid titer determination by ELISA, which was performed as described previously^29^. Briefly, serially diluted 20-hydroxyecdysone (20E) or the diluted culture media were incubated with anti-ecdysone antibody in the wells of an EIA plate coated with 20E-ovalbumin conjugate, followed by incubation with horseradish peroxidase-labeled secondary antibody.

### Whole-mount immunohistochemistry

Brains were dissected with surrounding tissues and fixed with 4% paraformaldehyde in phosphate-buffered saline for 2 h at room temperature. Immunohistochemistry was performed as described^12^ with following modifications: the incubation times with the primary and secondary antibodies were 24 h and 16 h, respectively, and an anti-BMS mouse monoclonal antibody was used as the primary antibody.

### Collection and processing of hemolymph for determination of myosuppressin (MS) titers

Hemolymph was sampled at 1-day intervals, starting from day 3 of wandering larva (W3) to day 7 of pupa (P7). Hemolymph was collected by cutting the anterior tip of the body and pressing the body of the animals, followed by the addition of sodium N, N’-diethyldithiocarbamate (5 mM), a phenoloxidase inhibitor, and centrifugation. The collected hemolymph (180 ml) was diluted with 180 μl of 2% acetic acid and 360 μl of methanol, and heated at 70 °C for 10 min. After cooling and centrifugation of the heated sample, 600 μl of the supernatant was transferred to a new tube and evaporated to about 100 μl residual volume using a vacuum centrifuge, followed by mixing with 900 μl of 0.1% trifluoroacetic acid (TFA). MS in the sample was extracted by solid-phase extraction using a Sep-Pak C8 cartridge (Waters). Elution of MS was performed using 40% acetonitrile containing 0.1% TFA. The eluate was freeze-dried and then dissolved in 150 μl of TBS containing 0.1% bovine serum albumin.

### Determination of MS titers in the hemolymph by TR-FIA

The wells of EIA plate (Costar, 3590) were incubated overnight at 4 °C with 50 μl of BMS-ovalbumin conjugate, followed by blocking with 200 μl of ‘BLOCK-ACE’ (Yukijirushi Megumilk, Hokkaido, Japan) for 1 h at 25 °C. After washing the wells, 50 μl of serially diluted BMS or test samples were distributed to the wells, followed by addition of 150 μl of 1: 200,000 diluted anti-BMS mouse antibody. After overnight incubation at 4 °C, the wells were washed and incubated with 50 μl of DELFIA Assay Buffer (Perkin Elmer) containing 1: 7,500 diluted biotin-labeled anti-mouse IgG antibody and 1: 500 diluted europium-labeled streptavidin (Perkin Elmer). After 1-h incubation at 25 °C, the wells were washed and added with 50 μl of DELFIA Enhancement Solution (Perkin Elmer). After 10-min incubation at 25 °C, the fluorescence was measured by a multimode plate reader ARVO X4 (Perkin Elmer).

### Molecular cloning of MS cDNA

Total RNA was extracted from 5 larval brains using RNeasy Mini Kit (QIAGEN). Single-stranded cDNA for PCR was synthesized using the total RNA and PrimeScript Reverse Transcriptase (TaKaRa). The following primers were designed from consensus sequences of previously determined MS cDNA sequences for *Mythimna unipuncta* and *Spodoptera littoralis*.

forward primer: 5′-AAGCCCTGGTCCGCGACTAC-3′

reverse primer: 5′-TAAGCCTGGTCGCTGCGTGTG-3′

PCR was performed under the following conditions: 94 °C for 2 min and 35 cycles of 94 °C for 30 sec, 60 °C for 30 sec, and 72 °C for 40 sec, with a final extension step of 7 min at 72 °C. The full-length cDNA sequence for *M. brassicae* MS was determined by 5′ and 3′ RACE as described previously^29^. The primers used were as follows.

5′ RACE GSP primer: 5′-TGTGGGTTGCGGTGGTTGGGCGTGGACG-3′

3′-RACE GSP primer: 5′-CAACTGCTCGACACCGGCATGAAGCGAC-3′

The nucleotide sequence of the *M. brassicae* MS cDNA is available in the DDBJ/EMBL/GenBank databases under the accession number LC180359. The signal sequence in the deduced amino acid sequence was predicted by the SignalP program ( http://www.cbs.dtu.dk/services/SignalP/).

### Quantitative RT-PCR

Total RNA was extracted from larval or pupal brains using the RNeasy Mini Kit. Synthesis of single-stranded cDNA and qRT-PCR were performed as described previously^29^. *RpL8* was used as an internal control for normalizing mRNA levels between stages. The primers used in this analysis were as follows.

*MS* forward primer: 5′-AAGCCCTGGTCCGCGACTAC-3′

*MS* reverse primer: 5′-TAAGCCTGGTCGCTGCGTGTG-3′

*RpL8* forward primer: 5′-ATCAAGGGTGTCGTGAAGGACATC-3′ *RpL8* reverse primer: 5′-CAGTAGACAAACTGGCCAGTGTAC-3′

### Statistic Analysis

Student t-test, One-way analysis of variance (ANOVA) and Tukey-Kramer multiple comparison test were used for statistic analysis.

## Additional Information

**How to cite this article:** Yamada, N. *et al*. Myosuppressin is involved in the regulation of pupal diapause in the cabbage army moth *Mamestra brassicae. Sci. Rep.* **7**, 41651; doi: 10.1038/srep41651 (2017).

**Publisher's note:** Springer Nature remains neutral with regard to jurisdictional claims in published maps and institutional affiliations.
